# Friedel-Craft Acylation of ar-Himachalene: Synthesis of Acyl-ar-Himachalene and a New Acyl-Hydroperoxide

**DOI:** 10.3390/molecules16075886

**Published:** 2011-07-14

**Authors:** Issam Hossini, Mohamed Anoir Harrad, Mustapha Ait Ali, Larbi El Firdoussi, Abdallah Karim, Pedro Valerga, M. Carmen Puerta

**Affiliations:** 1 Laboratoire de Chimie de Coordination, Faculté des Sciences-Semlalia BP 2390, Marrakech 40001, Morocco; Email: houssiniissam@yahoo.fr (I.H.); ma.harrad@yahoo.fr (M.A.H.); aitali@ucam.ac.ma (M.A.A.); elfirdoussi@ucam.ac.ma (L.E.F.); karim@ucam.ac.ma (A.K.); 2 Departamento de CMIM y Química Inorgánica, Facultad de Ciencias, Universidad de Cadiz, Campus Universitario Río San Pedro, Puerto Real 11510, Spain; Email: carmen.puerta@uca.es (M.C.P.)

**Keywords:** ar-himachalene, Friedel-Craft acylation, hydroperoxyde, hydrogen-bonds

## Abstract

Friedel-Craft acylation at 100 °C of 2,5,9,9-tetramethyl-6,7,8,9-tetrahydro-5*H*-benzocycloheptene [ar-himachalene (**1**)], a sesquiterpenic hydrocarbon obtained by catalytic dehydrogenation of α-, β- and γ-himachalenes, produces a mixture of two compounds: (3,5,5,9-tetramethyl-6,7,8,9-tetrahydro-5*H*-benzocyclohepten-2-yl)-ethanone (**2**, in 69% yield), with a conserved reactant backbone, and **3**, with a different skeleton, in 21% yield. The crystal structure of **3** reveals it to be 1-(8-ethyl-8-hydroperoxy-3,5,5-trimethyl-5,6,7,8-tetrahydronaphthalen-2-yl)-ethanone. In this compound O-H…O bonds form dimers. These hydrogen-bonds, in conjunction with weaker C-H…O interactions, form a more extended supramolecular arrangement in the crystal.

## 1. Introduction

**Figure 1:**
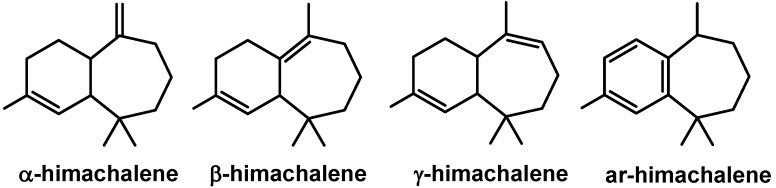
Main components of cedarwood oil.

Atlas Cedar (*Cedrus atlantica*) is the principal species in Moroccan forests. The tree plays a significant role in the socio-economy of Morocco, being good for furniture making. Furthemore, Atlas cedarwood oils are constituents of several products such as drugs, perfumes *etc*. [1,2,3,4,5,6]. The essential oil is mainly composed of the sesquiterpene hydrocarbons α-himachalene, β-himachalene, and γ-himachalene (Figure 1) which together can make up almost 70% of the composition [7,8] Ar-Himachalene (Figure 1), valued in perfumery [1,7] and the male-produced pheromone component of the flea beetle *Aphthona flava* [9,10], constitutes only 0.5% of the total oil.

Essential oils are an extremely useful source of starting materials for several industrial processes used for the synthesis of fragances and pharmaceutical compounds. A new route to high added value compounds from these cheap natural products is therefore a challenge, among which catalytic functionalization is of major interest. In fact, many different methods for functionalization of this essential oil were developed by our group within the framework of preparing new products having olfatory properties [11,12,13,14,15,16,17,18].

Several publications are devoted to the synthesis of new chiral ketone-peroxides, which play an important role in organic synthesis as substrates for the preparation of many classes of compounds such as chiral aromatic alcohols [19] or Schiff base [20] derivatives. As a contribution to this widespread area, we describe herein the synthesis and characterization of a new functionalized peroxide which could be a useful starting material for the preparation of new aromatic musk odorants [21,22].

## 2. Results and Discussion

In the first step of our procedure, ar-himachalene (**1**) was prepared in good yield by dehydrogenation of the mixture of α-, β- and γ-himachalene using the previously published method [23]. The reaction, catalyzed with Raney nickel, affords ar-himachalene in 93% yield. 

**Scheme 1:**
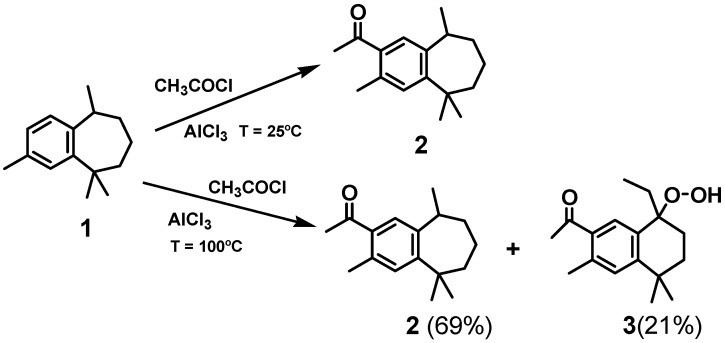
Friedel-Craft acylation of ar-himachalene.

In the second step of the procedure (Scheme 1), Friedel-Craft acylation of ar-himachalene with acetyl chloride at room temperature, using AlCl_3_ as catalyst, gave 1-(3,5,5,9-tetramethyl-6,7,8,9-tetrahydro-5H-benzocyclohepten-2-yl)-ethanone (**2**) as single product. When this reaction was carried out at 100 °C, keeping other conditions constant, **2** as major product (69%) and 1-(8-ethyl-8-hydroperoxy-3,5,5-trimethyl-5,6,7,8-tetrahydro-naphthalen-2-yl)-ethanone (**3**, in 21% yield) were obtained. 

The spectroscopic data of the two newly prepared products **2** and **3** are consistent with the assigned structures, characterized in ^1^H-0 and ^13^C-NMR spectroscopies by the existence of only two C*H* aromatic signals, which appeared as singlets, at 7.10 ppm and 7.50 ppm for **2** and at 7.09 ppm and 7.71 ppm for **3**. The methyl moiety of the acetyl groups appeared at 2.50 ppm and 2.48 ppm for **2** and **3**, respectively. The rearrangement of the seven membered ring in compound **3**, is shown by the presence of ethyl group signals at 0.85 ppm (CH_2_C*H*_3_, d, *J* = 7.5 Hz) and 1.85 ppm (C*H*_2_CH_3_, q, *J* = 7.5 Hz). These assignments are in agreement with the ^13^C-NMR data. In the ^1^H-NMR of compound **3** a signal at 2.12 ppm attributable to OO*H* was found. Its relative sharpness points out the participation of the hydroperoxide group in hydrogen-bonds. 

**Scheme 2:**
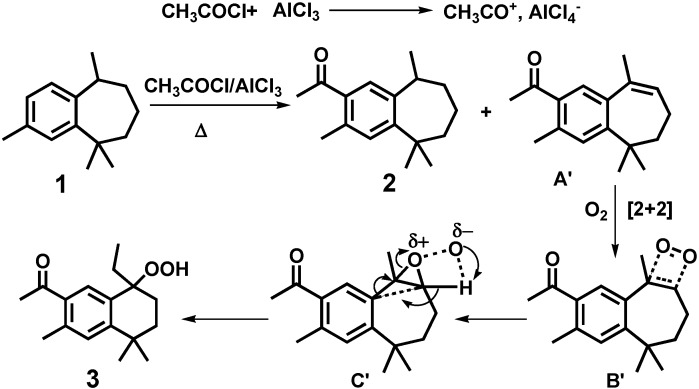
A possible mechanism for the formation of acyl-hydroperoxide (3).

The formation of this new acyl-hydroperoxyde **3** can be explained by the oxyfunctionalization of acyl-ar-himachalene with molecular oxygen when exposed to air, as described by Orfanopoulos *et al*. [24]. Scheme 2 shows the proposed mechanism. The molecular oxygen [2 + 2] ene reaction of the product A’ (probably formed *in situ* at 100 °C as a consequence of the basicity of AlCl_4_^−^) gives intermediate B’, which was converted to the transition state C’ [24]. Rearrangement of the transition state C’ gives the hydroperoxide **3**. We note here that the derivatives of himachalene with seven-membered rings are susceptible to undergo rearrangements. Recently, Gouygou *et al*. [25,26] have reported the reactivity of epoxides derived from the sesquiterpenic himachalenes in the presence of Lewis or Bronsted acids which catalyze the rearrangement of the ring-opening reactions to give ketones or aldehydes through the formation of carbocations.

**Figure 2:**
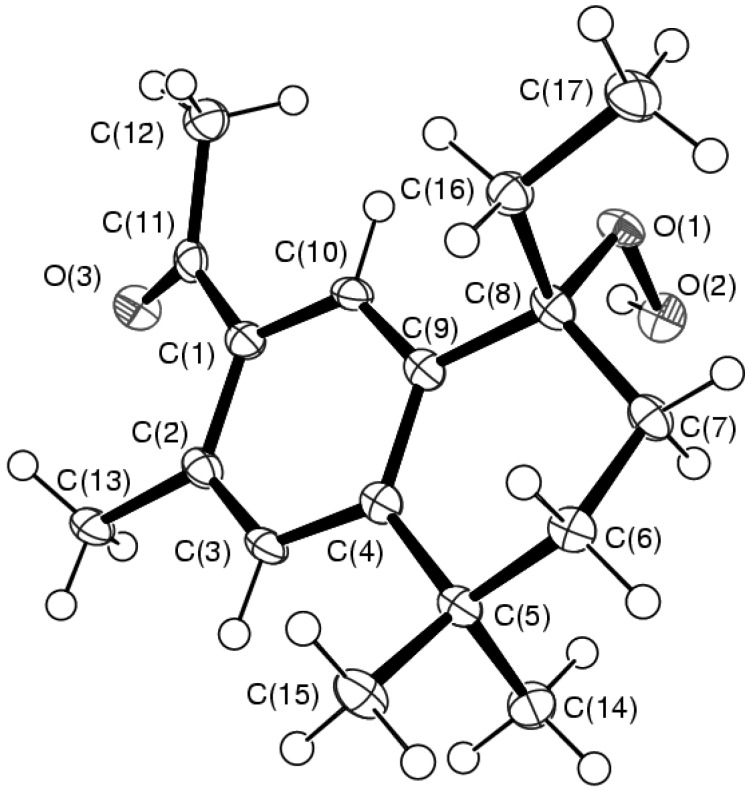
**ORTEP** drawing (50 % thermal ellipsoids) of **3**.

The X-ray single crystal structure analysis permits us to confirm the structure of the rearranged product **3** (Figure 2). Selected bond lengths (Å) and angles (°) are given in Table 1.

**Table 1:** Selected bond lengths (Å) and angles (°) for **3** (e.s.d.s in parentheses).

O(1)-O(2)	1.462(3)	O(1)-C(8)-C(9)	109.8(2)
O(1)-C(8)	1.452(3)	O(1)-C(8)-C(16)	102.4(2)
O(3)-C(11)	1.232(3)	O(1)-C(8)-C(7)	110.2(2)
O(2)-O(1)-C(8)	108.73(19)	O(3)-C(11)-C(1)	120.8(3)

**Figure 3:**
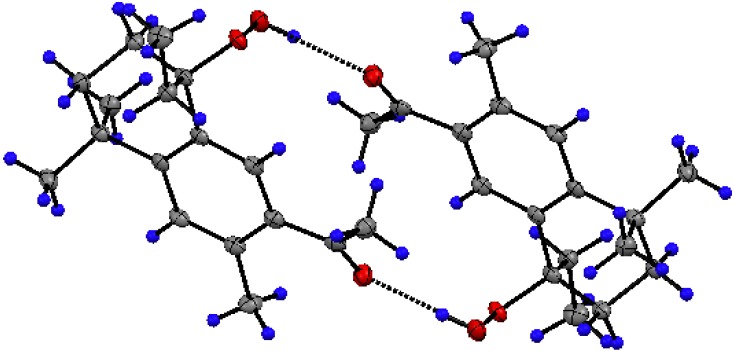
Molecular dimers formed by two hydrogen-bonds with a total bond length between donor O(2) and acceptor O(3) of 2.765(3) Å.

The formation from ar-himachalene of a new bicyclic 1,2,3,4-tetrahydronaphthalene is observed. Although the C(8) asymmetric center has the *R*-configuration in the molecule shown in Figure 2, the existence of a center of symmetry in space group P2_1_/n corroborates the formation of a racemic mixture. A bond length of 1.462(3) Å has been found for the hydroperoxide, corresponding to a single bond O-O. It compares well with other hydroperoxides [27,28,29,30,31]. Bond lengths and angles around C(8) show its sp^3^ character. On the other hand, the environment of C(11) in the acyl group shows its usual sp^2^ geometry. In addition to demonstrating the nature of these functional groups and the new skeleton with two six membered condensed rings instead of ar-himachalene, the crystal structure also reveals the existence of intra- and intermolecular hydrogen-bonds (Table 2). The strongest of these interactions contributes to form dimers (Figure 3).

**Table 2:** Hydrogen-Bonds (lengths in Å, angles in °) for **3** (e.s.d.s in parentheses).

D--H..A	d(D--H)	d(H..A)	d(D....A)	angle (D--H..A)	Symmetry label
O(2)--H(2A)..O(3)	0.8400	1.9400	2.765(3)	165.00	3_556
C(3)--H(3)..O(1)	0.9500	2.4800	3.321(4)	147.00	4_555
C(7)--H(7B)..O(2)	0.9900	2.3700	2.806(4)	106.00	intramolecular

The association of two molecules is also enhanced by a C(7)--H(7B)..O(2) intramolecular interaction. The dimer adopts an overall conformation in which the rings are oriented parallel to each other. 

**Figure 4:**
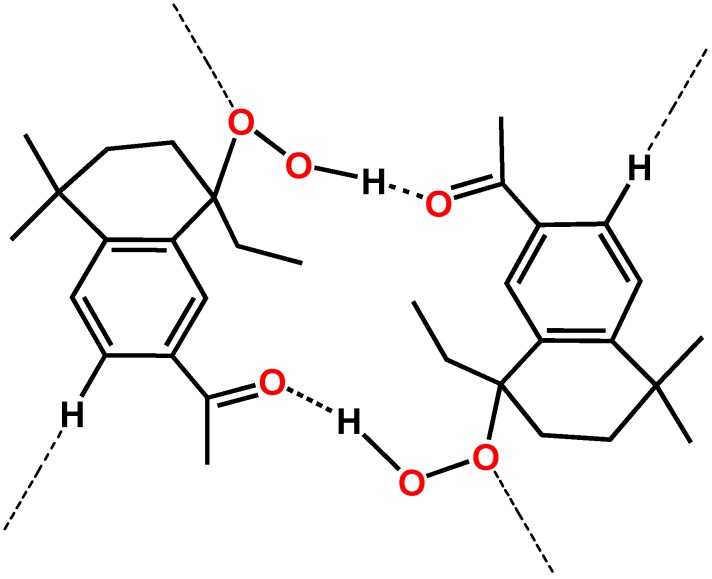
Hydrogen-bond patterns.

A second weaker intermolecular hydrogen-bond, C(3)—H…O(1) 3.321(4) Å, is responsible for the interaction between the first one and other dimers forming chains of indefinite length. In this hydrogen-bond the internal hydroperoxide oxygen atom O(1) acts as acceptor. The whole hydrogen-bonded scheme involves most of all potential proton donors and acceptors. An useful way to define a hydrogen-bond pattern is the graph set [32]. In our case, the morphology of hydrogen-bonded array is shown in Figure 4, where the intramolecular hydrogen-bond has been suppressed for clarity.

**Figure 5:**
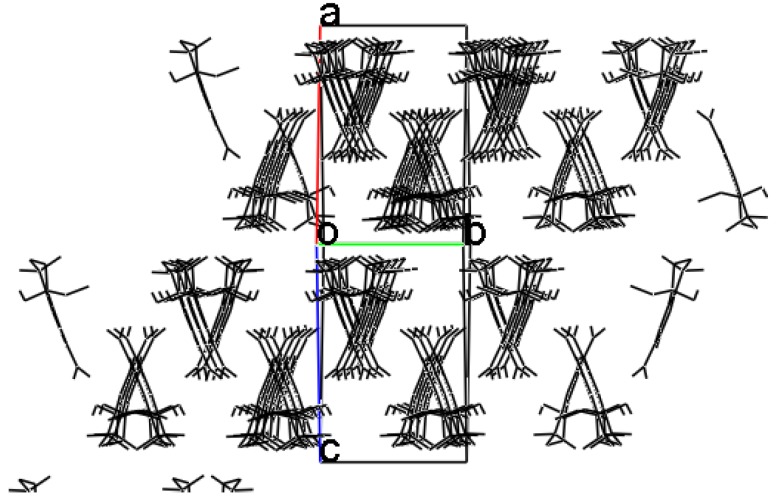
Layers of molecular rows aligned to the (−1 0 1) plane.

Through this interaction each dimer is bonded to four other bimolecular sets. The global effect of all the described intermolecular interactions is a supramolecular arrangement in columns. These rows form layers aligned to the (−1 0 1) plane (Figure 5).

## 3. Experimental

### 3.1. General

NMR studies were performed on a Bruker Avance 300 spectrometer in CDCl_3_, chemicals shifts are given in ppm relative to external TMS and coupling constant (*J*) in Hz. Liquid chromatography was performed on silica gel (Merk 60, 220-440 mesh; eluent: hexane/ethylacetate). All the reagents and solvents used in the experiments were purchased from commercial sources as received without further purification (Aldrich, Fluka, Acros), and Ar-himachalene were prepared by our team [23].

### 3.2. General Acylation Procedure:

Acyl chloride (1.56 mmol, 0.122 g) and AlCl_3_ (1.87 mmol, 0.25 g) were mixed in nitromethane (10 mL), and heated at 100 °C. The aromatic compound (1 mmol) was added to the mixture and the progress of the reaction was monitored by TLC. At the end of the reaction, the solvent was evaporated on a rotary evaporator and a saturated solution of NaHSO_4_ (20 mL) was added. The crude material was extracted with Et_2_O (3 × 10 mL) and dried over anhydrous MgSO_4_. Evaporation of the solvent afforded the crude products which were separated by column chromatography using hexane/ethyl acetate (8/2). The products were obtained in yields of 69% for **2** and 21% for isolated **3**.

### 3.3. Acyl-Ar-Himachalene ***2***:

Pale yellow oil, NMR ^1^H (CDCl_3_), δ ppm: 1.24 (3 H, s, C*H*_3_), 1.32 (3 H, d, *J* = 6.9, C*H*_3_), 1.36 (3 H, s, C*H*_3_), 1.40–1.45 (1 H, m, C*H*H), 1.54–1.57 (1 H, m, CH*H*), 1.67–1.74 (4 H, m, C*H*_2_), 2.44 (3 H, s, C*H*_3_), 2.48 (3 H, s, ArC*H*_3_), 3.20 (1 H, m, C*H*CH_3_), 7.14 (1 H, s, C4_ar_-*H*), 7.49 (1 H, s, C1_ar_-*H*); NMR ^13^C (CDCl_3_): 19.82, 20.61, 22.73, 27.95, 28.54, 32.67, 33.21, 35.24, 38.61, 39.74, 125.97, 129.57, 133.70, 134.98, 140.03, 150.42, 198.79.

### 3.4. Acyl-Hydroperoxyde ***3***:

White solid, ^1^H-NMR (CDCl_3_), δ ppm: 0.85 (3 H, t, *J* = 7.5 Hz, CH_2_C*H*_3_), 1.20 (3 H, s, C(C*H*_3_)_2_), 1.22 (3 H, s, C(C*H*_3_)_2_), 1.55–1.65 (1 H, m, *H*CH), 1.71–1.74 (1 H, m, HC*H*), 1.81 (2 H, q, *J* = 7.5 Hz, C*H*_2_CH_3_), 1.87–1.97 (1 H, m, *H*CH), 2.12 (1 H, s, OO*H*), 2.25–2.39 (1 H, m, HC*H*), 2.43 (3 H, s, C3_ar_-C*H*_3_), 2.47 (3 H, s, COC*H*_3_), 7.09 (1 H, s, C4_ar_-*H*), 7.72 (1 H, s, C1_ar_-*H*); ^13^C-NMR (CDCl_3_): 8.62, 21.85, 25.23, 29.31, 31.04, 31.33, 32.19, 34.16, 35.30, 84.65, 128.34, 130.24, 133.70, 134.40, 138.03, 151.28, 199.50.

### 3.5. X-ray Structure Determination

Single crystals of compound **3** were obtained and mounted on glass fiber to carry out the crystallographic study. Crystal data and experimental details are given in Table 3. X-ray diffraction data collection was measured at 100 K on a Bruker Smart APEX CCD 3-circle diffractometer using a sealed tube source, graphite monochromated Mo Kα radiation (λ = 0.71073 Å) at the Servicio Central de Ciencia y Tecnología de la Universidad de Cádiz. Four sets of frames were recorded over a hemisphere of the reciprocal space by omega scan with δ(ω) 0.30 degrees and exposure of 10 seconds per frame. Correction for absorption was applied by scans of equivalents using program SADABS [33]. An insignificant crystal decay correction was also applied. The structure was solved by direct methods, completed by subsequent difference Fourier syntheses and refined on F2 by full matrix least-squares procedures using the programs contained in SHELXTL package [34] Non hydrogen atoms were refined with anisotropic displacement parameters. The program ORTEP-3 was used for plotting [35]. Crystallographic data in CIF format for **3** have been deposited at Cambridge Crystallographic Data System with reference CCDC 752508.

**Table 3:** Crystal Data and Details of the Structure Determination for Compound **3**.

Formula	C_17_ H_24_ O_3_
Formula Weight	276.36
Crystal System	Monoclinic
Space group	P2_1_/n (No. 14)
a, b, c (Å)	14.459(9), 8.166(5), 14.459(9)
α, β, γ (°)	90, 115.50(2), 90
V (Å^3^)	1540.9(17)
Z	4
d(calc) (g/cm^3^)	1.191
µ (MoKα) (mm^−1^)	0.080
F(000)	600
Crystal Size (mm)	0.27 × 0.54 × 0.56
Temperature (K)	100
Radiation, λ (Å)	MoKα, 0.71073
θ Min-Max (°)	1.7, 25.0
Dataset (h, k, l ranges)	−17:16; −9:7; −17:17
Tot., Uniq. Data, R(int)	7525, 2657, 0.188
Observed data [I > 2.0 σ(I)]	2236
Nref, Npar	2657, 187
R, wR_2_, S *	0.0703, 0.1937, 1.06
Max. and Av. Shift/Error	0.000, 0.000
Min. and Max. Resd. Dens. (e/A^3^)	−0.31, 0.31

* w = 1/[σ^2^(Fo^2^) + (0.0755P) ^2^ + 0.9681P] where P = (Fo^2^ + 2Fc^2^)/3

## 4. Conclusions

Following our studies on the suitable use of natural resources in order to obtain products of high added value, in the present work we have shown that the Friedel-Crafts acylation of ar-himachalene, under mild conditions, at 25 °C, produces (3,5,5,9-tetramethyl-6,7,8,9-tetrahydro-5*H*-benzocyclohepten-2-yl)-ethanone (**2**). However, at 100 °C, the Friedel-Crafts reaction leads to a mixture of two compounds: (3,5,5,9-tetramethyl-6,7,8,9-tetrahydro-5*H*-benzocyclohepten-2-yl)-ethanone (**2**, 69%), in which the backbone of reactant is conserved, and 1-(8-ethyl-8-hydroperoxy-3,5,5-trimethyl-5,6,7,8-tetrahydronaphthalen-2-yl)-ethanone (**3**, 21%). In the solid state this new hydroperoxy compound forms dimers by hydrogen-bonds. These dimers are grouped in extended pattern by weaker hydrogen-bonds.
